# Application of texture analysis to DAT SPECT imaging: Relationship to clinical assessments

**DOI:** 10.1016/j.nicl.2016.02.012

**Published:** 2016-02-23

**Authors:** Arman Rahmim, Yousef Salimpour, Saurabh Jain, Stephan A.L. Blinder, Ivan S. Klyuzhin, Gwenn S. Smith, Zoltan Mari, Vesna Sossi

**Affiliations:** a Department of Radiology, Johns Hopkins University, Baltimore, MD, United States; b Department of Electrical and Computer Engineering, Johns Hopkins University, Baltimore, MD, United States; c Department of Neurology and Neurosurgery, Johns Hopkins University, Baltimore, MD, United States; d Center for Imaging Science, Johns Hopkins University, Baltimore, MD, United States; e Pacific Parkinson's Research Centre, University of British Columbia, Vancouver, Canada; f Department of Physics & Astronomy, University of British Columbia, Vancouver, Canada; g Department of Psychiatry and Behavioral Sciences, Johns Hopkins University, Baltimore, MD, United States

**Keywords:** DAT SPECT, Heterogeneity, Textural features, Disease progression, Parkinson's disease

## Abstract

Dopamine transporter (DAT) SPECT imaging is increasingly utilized for diagnostic purposes in suspected Parkinsonian syndromes. We performed a cross-sectional study to investigate whether assessment of texture in DAT SPECT radiotracer uptake enables enhanced correlations with severity of motor and cognitive symptoms in Parkinson's disease (PD), with the long-term goal of enabling clinical utility of DAT SPECT imaging, beyond standard diagnostic tasks, to tracking of progression in PD. Quantitative analysis in routine DAT SPECT imaging, if performed at all, has been restricted to assessment of mean regional uptake. We applied a framework wherein textural features were extracted from the images. Notably, the framework did not require registration to a common template, and worked in the subject-native space. Image analysis included registration of SPECT images onto corresponding MRI images, automatic region-of-interest (ROI) extraction on the MRI images, followed by computation of Haralick texture features. We analyzed 141 subjects from the Parkinson's Progressive Marker Initiative (PPMI) database, including 85 PD and 56 healthy controls (HC) (baseline scans with accompanying 3 T MRI images). We performed univariate and multivariate regression analyses between the quantitative metrics and different clinical measures, namely (i) the UPDRS (part III - motor) score, disease duration as measured from (ii) time of diagnosis (DD-diag.) and (iii) time of appearance of symptoms (DD-sympt.), as well as (iv) the Montreal Cognitive Assessment (MoCA) score. For conventional mean uptake analysis in the putamen, we showed significant correlations with clinical measures only when both HC and PD were included (Pearson correlation *r* = − 0.74, p-value < 0.001). However, this was not significant when applied to PD subjects only (*r* = − 0.19, p-value = 0.084), and no such correlations were observed in the caudate. By contrast, for the PD subjects, significant correlations were observed in the caudate when including texture metrics, with (i) UPDRS (p-values < 0.01), (ii) DD-diag. (p-values < 0.001), (iii) DD-sympt (p-values < 0.05), and (iv) MoCA (p-values < 0.01), while no correlations were observed for conventional analysis (p-values = 0.94, 0.34, 0.88 and 0.96, respectively). Our results demonstrated the ability to capture valuable information using advanced texture metrics from striatal DAT SPECT, enabling significant correlations of striatal DAT binding with clinical, motor and cognitive outcomes, and suggesting that textural features hold potential as biomarkers of PD severity and progression.

## Highlights

• Aim to enable image-based tracking of progression in Parkinson's disease
• Texture analysis of clinical dopamine transporter (DAT) SPECT images (DaTscans)
• Significant correlations with clinical, motor and cognitive outcomes

## Graphical abstract

Improved correlation of heterogeneity metrics (vs. conventional mean-uptake) against clinical assessments.

**Figure:**
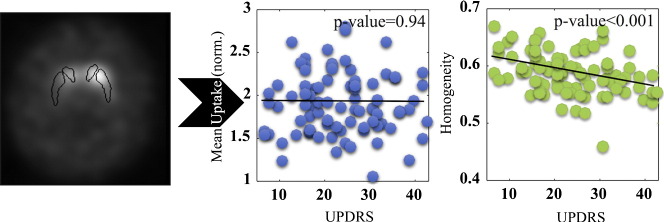

## 1 Introduction

Imaging of the dopaminergic system with SPECT has become widespread in Europe and has entered a new active phase in the US since ^123^I-ioflupane-dopamine transporter (DAT) SPECT was approved by the FDA in 2011 (Catafau and Tolosa, 2004, Grachev et al., 2012, Kupsch et al., 2012).

For diagnosis, visual interpretation of DAT SPECT images has been the common assessment approach (Catafau and Tolosa, 2004, Grachev et al., 2012, Kupsch et al., 2012). Meanwhile, more objective assessment can be performed with quantitative analysis (Djang et al., 2012) involving manual or automated ROI drawing and analysis of mean-ROI uptake (Badiavas et al., 2011, Koch et al., 2005). Quantitative analysis may be more sensitive to detecting the early stages of disease and to better track disease progression. Such an effort is also consistent with the aim of the Parkinson's Progressive Marker Initiative (PPMI) (Parkinson Progression Marker, 2011) to identity biomarkers of PD progression, a critical step in the development of novel and enhanced treatments for PD.

In the present work, we perform a cross-sectional study to investigate whether use of advanced textural features enables enhanced correlations with clinical assessments. This is a step towards the long-term goal of enabling clinical utility of DAT SPECT imaging, beyond standard diagnostic tasks, to tracking of progression in PD. Our proposed approach is based on the observation that SPECT and PET images convey important information at the voxel level, whereas commonly used regions-of-interest (ROI) mean uptake analysis may oversimplify the available spatial uptake information. We aim to explore a texture quantification paradigm applied to SPECT neurochemical imaging. The present work includes assessment of both motor and non-motor symptoms, since it has been shown that a number of neuropsychiatric symptoms and cognitive disorders are more common in PD compared to the general population and contribute to the disability associated with the illness (de la Riva et al., 2014, Gustafsson et al., 2015).

There is emerging literature on the use of advanced metrics that quantify tumor uptake heterogeneity and their enhanced prediction of treatment response and survival outcome in different cancers (Aerts et al., 2014, Asselin et al., 2012, Chicklore et al., 2013, Eary et al., 2008, El Naqa et al., 2009, Hatt et al., 2015, Kumar et al., 2012, Lambin et al., 2012, Rahmim et al., 2016, Tixier et al., 2014, Tixier et al., 2011, van Velden et al., 2011, Vriens et al., 2012). We have, in the past, investigated advanced texture analysis in the context of quantitative brain PET imaging, in studies of PD (Gonzalez et al., 2013, Klyuzhin et al., 2015, Sossi et al., 2012) and neuroinflammation (Rahmim et al., 2012). These techniques have the advantage of not requiring normalization/registration of ROIs to a common structure. In the present work, we focus on DAT SPECT imaging, given its increasingly popular clinical usage. Furthermore, we have strong evidence that application of texture metrics from the higher resolution spectrum of PET images to lower resolution imaging in the domain of SPECT can retain significant information (Blinder et al., 2014).

PD is a progressive, degenerative movement disorder. It is characterized by dopaminergic neuron loss in the substantia nigra with the loss of neuron terminals in basal ganglia structures, particularly the dorsal striatum (composed mainly of the putamen and dorsal part of the caudate) (Brooks et al., 1990, Garnett et al., 1987, Stoessl et al., 2011). Pathophysiologic studies of dopamine loss have in fact clearly indicated highly heterogeneous uptake (primarily in the form of sharp rostrocaudal and dorsoventral gradients) in the caudate and the putamen (see Fig. 1 in Kish et al., 1988). We hypothesize that application of textural features will improve the ability to capture state of disease as manifested in the form of uneven loss of tracer uptake within these structures. Textural information, we postulate, can thus provide improved correlations with motor and non-motor outcomes in PD patients.

## 2 Materials and methods

### 2.1 DAT SPECT images

We analyzed DAT scan images from the PPMI database (www.ppmi-info.org/data) (Initiative, P.s.P.M., 2012, Parkinson Progression Marker, 2011). All scans selected were performed at baseline. For consistency our analysis only included participants who had SPECT data acquired on similar kinds of scanner (Siemens, 2-headed ECAM or Symbia systems), and who had additionally undergone a high-resolution 3 T MRI scan. With these selection criteria, we arrived at 141 subjects, which included 85 PD and 56 HC.

The subjects in the database were imaged 4 ± 0.5 h following injection of 111-185 MBq of DAT SPECT (^123^I-Ioflupane). Subjects were pretreated with saturated iodine solution (10 drops in water) or perchlorate (1000 mg) prior to the injection to block thyroid update. Raw projection data were acquired into a 128 × 128 matrix stepping each 3 deg. for a total of 120 projections into two 20% symmetric photopeak windows centered on 159 keV and 122 keV with a total scan duration of approximately 30–45 min. The SPECT raw projection data were imported to a HERMES (Hermes Medical Solutions, Stockholm, Sweden) system for iterative OSEM reconstruction. This was done for all studies to ensure consistency of the reconstruction method.

The reconstructed files were then transferred to PMOD (PMOD Technologies, Zurich, Switzerland) for subsequent processing. Attenuation correction ellipses where drawn on the images and Chang 0 attenuation correction was applied to the images utilizing a site specific mu that was empirically derived from phantom data acquired during site initiation for the trial. Once attenuation correction was completed, a standard 3D Gaussian filter (6.0 mm FWHM) was applied.

### 2.2 Image analysis and quantification paradigm

We first segmented the high-resolution MRI images to obtain the boundaries of the caudate and putamen (both left and right), as well as the occipital cortex (used as a reference region), utilizing a multi-atlas segmentation method (Tang et al., 2013). We also resampled each SPECT image onto the corresponding MRI grid performing rigid mapping, using the FSL utility FLIRT (Jenkinson and Smith, 2001).

We subsequently computed mean radiotracer concentration in each ROI and divided it by the concentration in the reference region, to obtain an approximate estimate of the distribution volume ratio, conventionally used as a quantitative outcome (Badiavas et al., 2011, Djang et al., 2012, Koch et al., 2005). Since PD typically affects the striata in an asymmetric fashion, the more and less affected sides were considered separately in subsequent analysis.

For our proposed analysis, we performed Haralick analysis, which has found increasing utility in the field of radiomics and heterogeneity quantification. This is especially because Haralick analysis captures valuable local information, and at the same time, some of its metrics have been shown to depict very good robustness to segmentation (Echegaray et al., 2015) and overall test–retest reproducibility (Grkovski et al., 2015, Leijenaar et al., 2013), even outperforming conventional mean uptake analysis (Tixier et al., 2012, van Velden et al., 2016), which we presumed would be advantageous for tracking of disease progression. We computed and evaluated thirteen Haralick texture measures: (1) energy, (2) entropy, (3) correlation, (4) contrast (also known as inertia (Conners and Harlow, 1980, Oh et al., 1999)), (5) variance, (6) sum mean, (7) agreement (Parkkinen et al., 1990) (also known as Cohen's kappa (Cohen, 1960)), (8) cluster shade, (9) cluster tendency (or prominence), (10) homogeneity, (11) max probability, (12) inverse variance, and (13) dissimilarity.

We note here that Martinez-Murcia et al. (2014) utilized 11 of these Haralick texture features (excluding agreement and dissimilarity) in a recent study. However, that study only focused on automated *diagnosis* of PD, i.e. the ability of a metric to discriminate between control and affected subjects, unlike the present work, which significantly changes focus to correlating imaging measures with motor and non-motor symptoms. Furthermore, comparisons with conventional analysis were not reported by the authors. Here, we aim to identify the added value of imaging measures with respect to conventional analysis, in a completely different paradigm of correlation with clinical assessments, aiming ultimately to identify imaging biomarkers of disease progression.

As prerequisite for computation of Haralick metrics, we extracted the gray-level co-occurrence matrix (GLCM) (Conners et al., 1984, Haralick et al., 1973). A 32 Gy-level quantization was utilized, and 13 spatial directions in 3D were considered, with voxels separated by a distance of 1, and the 13 matrices averaged and subsequently normalized. Modifying quantization bins and distance was not seen to significantly alter relative performance of metrics, with the exception of inverse variance that was highly modulated.

### 2.3 Correlation with clinical measures

We performed Pearson correlation analysis between the above-mentioned image-based metrics and the following clinical measures: (i) The unified Parkinson's disease rating scale (UPDRS) – part III (motor). (ii, iii) Disease duration (DD), taken with respect to time of diagnosis (DD-diag.) as well as time of appearance of symptoms (DD-sympt.). Finally, we performed analysis involving a non-motor, cognitive outcome, specifically (iv) the Montreal Cognitive Assessment (MoCA).

### 2.4 Statistical analysis

Univariate correlation was first performed (Pearson correlation). Correction for multiple testing of different features (metrics) was performed using the false discovery rate (FDR) Benjamini–Hochberg (BH) step-up procedure. This procedure works as follows: (i) We order the *k* = 1… *m* tested variables according to their p-values in increasing order (denoted *P*_(1)_… *P*_(*m*)_). (ii) For a given *α* (we set, *α* = 0.05), we find the largest *k* satisfying $P_{\left(k\right)}\leq \frac{k}{m}\alpha$. (iii) Positive discoveries are declared for tested variables corresponding to *P*_(1)_… *P*_(*k*)_.

Following univariate analysis, multivariate stepwise linear regression analysis was also performed to identify independent factors. Metrics were entered sequentially (if p-value < 0.10) and then removed if they became non-significant (if p-value > 0.05). In addition to conventional and proposed textural features, subject age was also included within the analysis to take into account any confounding effects.

## 3 Results

**Fig. 1:**
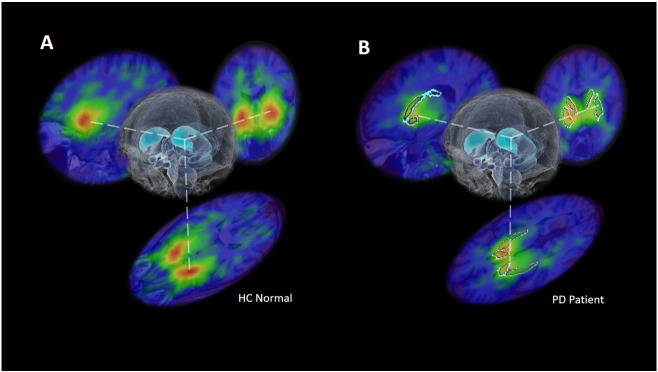
Examples of transaxial, coronal and sagittal slices through the DaT SPECT images for a HC subject (A) and a PD subject (B), also showing segmentation for caudate and putamen (B).

Fig. 1 shows example SPECT images for HC and PD subjects, and an overlaid MR-based segmentation, following SPECT-MRI registration as described in the Materials and methods section.

**Fig. 2:**
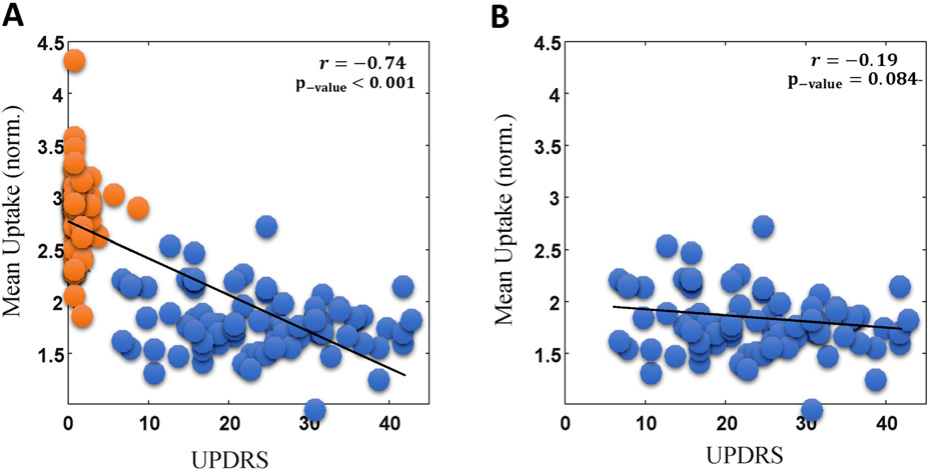
Plots of normalized mean ROI uptake (y-axis) vs. UPDRS, when including both PD (n = 85; blue ‘o’) and HC (n = 56; orange) (A), and when only including PD subjects (B). The results are shown for the more affected putamen side.

When both HC and PD subjects were included in correlation analysis, conventional normalized mean uptake approach resulted in significant correlations with clinical measures. For instance, Fig. 2(A) depicts significant correlation (Pearson correlation *r* = − 0.74, p-value < 0.001) between mean uptake and UPDRS score for the more affected putamen side. However, we found that the correlation became insignificant when HCs were removed from the dataset as clearly shown in Fig. 2(B) (*r* = − 0.19, p-value = 0.084). For further confirmation, we also utilized conventional mean uptake values within the PPMI database (generated using a different image analysis pipeline than ours), and made similar observations of no significant correlation in the PD-only case (*r* = − 0.20, p-value = 0.065). Furthermore, very similar patterns were observed when correlating against disease duration (both DD-diag. And DD-sympt.) (not shown).

**Fig. 3:**
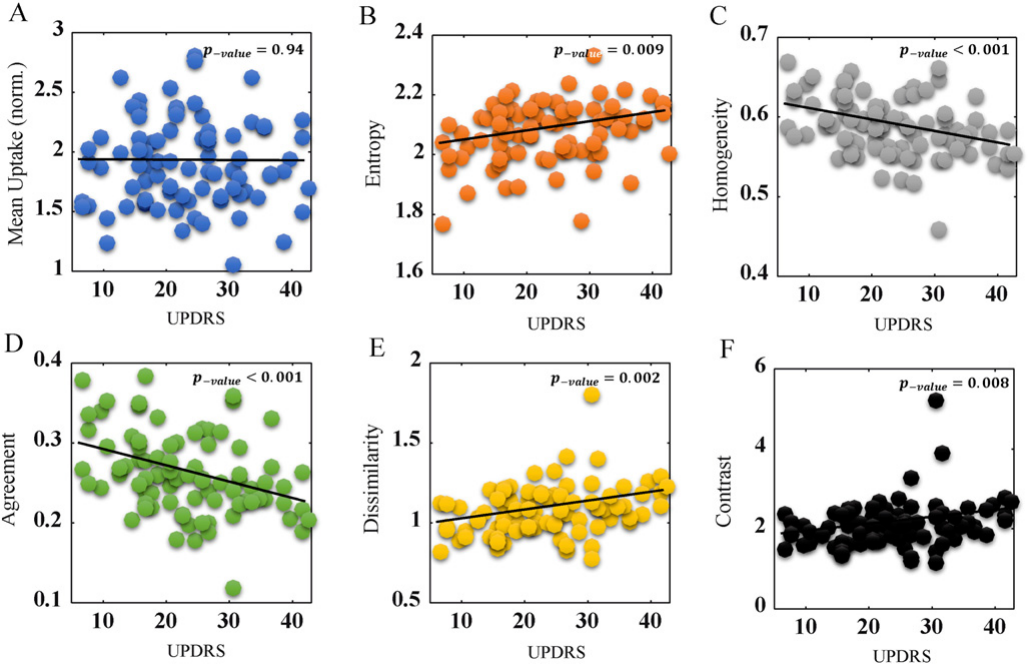
Plots of metric values vs. UPDRS, for conventional mean uptake (A), as well as five Haralick texture metrics (B-F). The correlation values were − 0.008, 0.28, − 0.36, − 0.37, 0.33 and 0.28. The more affected caudate side is shown, for PD subjects (n = 85).

Our key finding in this work has been that some of the advanced textural features, that are also pronounced in emerging radiomics/texture-analysis research in cancer imaging (Hatt et al., 2015), depict significantly enhanced correlations with clinical measures within the PD population compared to traditional measures. Interestingly, the caudate was seen to exhibit the highest correlation with severity of motor and cognitive symptoms (see also Discussion section). Fig. 3 shows correlation patterns against UPDRS for a number of metrics in the caudate (more affected side). Conventional mean uptake depicted no correlation (p-value = 0.94) (as we also observed using the PPMI database image analysis pipeline; p-value = 0.75; not shown). By contrast, when utilizing entropy, homogeneity, agreement, dissimilarity and contrast, p-values of 0.0092, < 0.001, < 0.001, 0.0022 and 0.0085 were obtained, respectively, as shown in the figure. Pearson correlations (*r*) are also reported in the plot caption. The signs of the correlations for the 5 texture metrics (+,−,−,+,+ respectively) were all consistent with their specific definitions, since the metrics homogeneity and agreement decrease in value with increasing heterogeneity in tracer uptake, unlike entropy, dissimilarity, and contrast. Following correction for multiple testing (FDR adjustments), all five metrics retained statistical significance (Table 1).

**Fig. 4:**
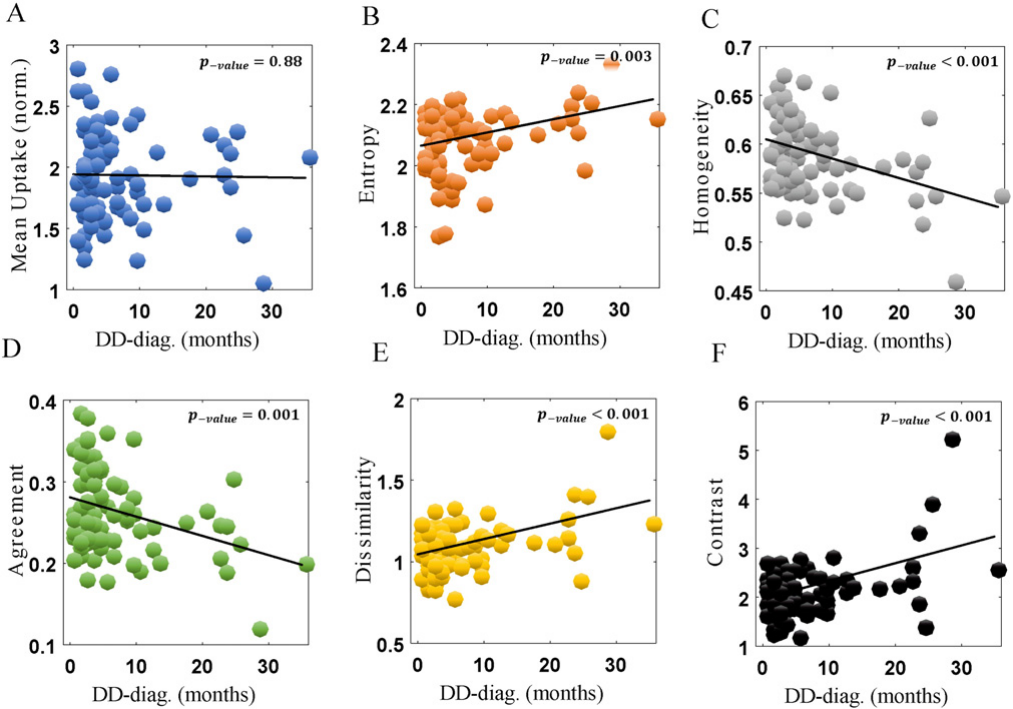
Plots of metric values vs. DD (from diagnosis), for conventional mean uptake (A), as well as five Haralick texture metrics (B-F). The correlation values were − 0.016, 0.32, − 0.39, − 0.35, 0.44 and 0.47. The more affected caudate side is shown, for PD subjects (n = 85).

When correlating against disease duration as measured from time of diagnosis (DD-diag.), as shown in Fig. 4, even more pronounced effects were observed. Unlike conventional analysis (p-value = 0.88), the same features as in Fig. 3, i.e. entropy, homogeneity, agreement and dissimilarity, and contrast, depicted very significant p-values (0.0027, < 0.001, 0.0012, < 0.001 and < 0.001), all of which were also statistically significant after FDR adjustments (Table 1).

**Fig. 5:**
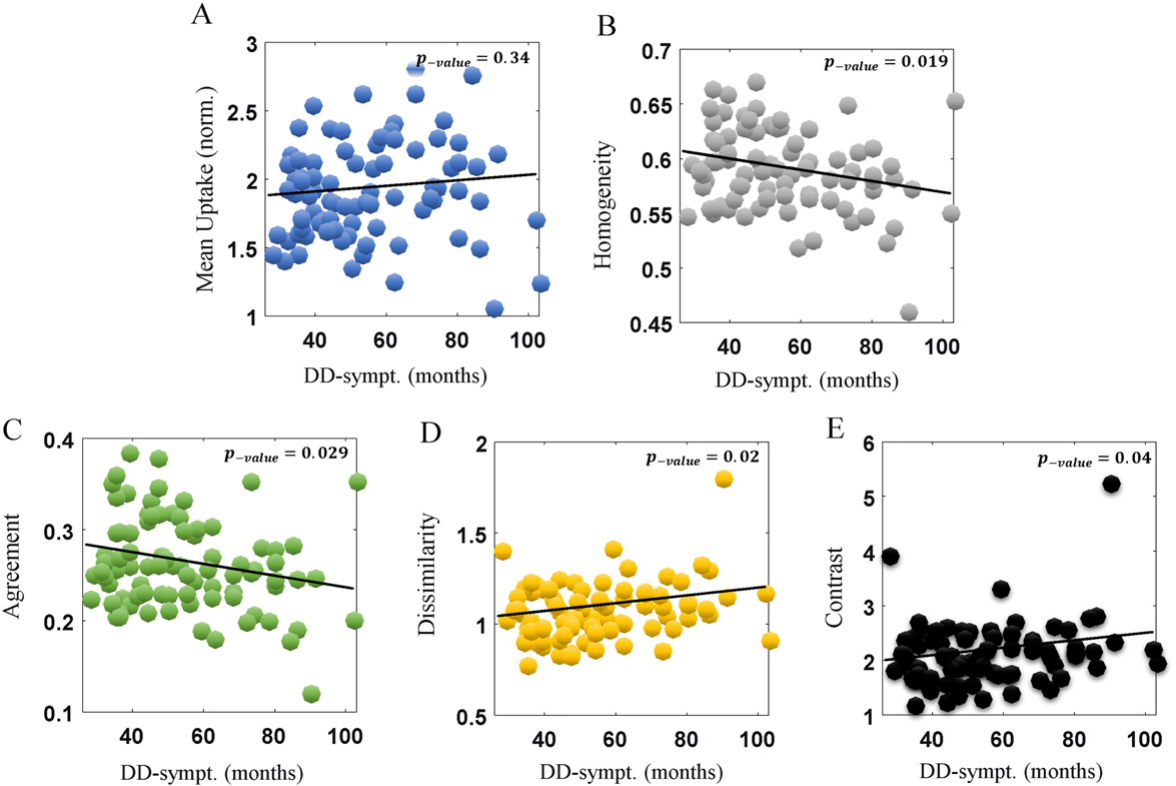
Plots of metric values vs. DD (from symptoms), for conventional mean uptake (A), as well as four Haralick texture metrics (B–E). The correlation values were 0.11, − 0.25, − 0.24, 0.25 and 0.22. The more affected caudate side is shown, for PD subjects (n = 85).

Similar patterns were observed when considering disease duration as measured from time of appearance of symptoms (DD-sympt.), as shown in Fig. 5. Specifically, as before, conventional mean uptake depicted no correlation (p-value = 0.34), whereas four metrics (homogeneity, agreement, dissimilarity and contrast) exhibited significant correlations, with p-values of 0.019, 0.029, 0.021 and 0.040 respectively, though they were not retained after FDR adjustments. The signs of the correlations for texture metrics both Fig. 4, Fig. 5 were again consistent with metric interpretations as in Fig. 3.

**Fig. 6:**
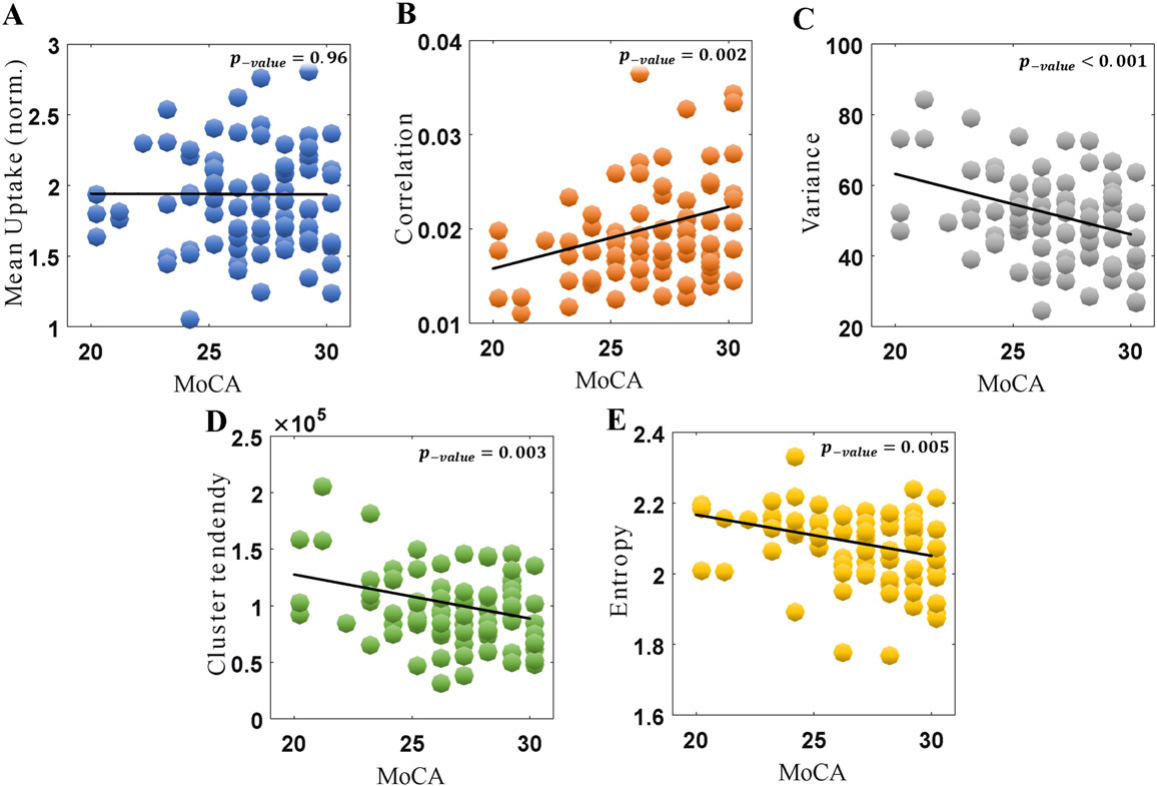
Plots of metric values vs. MoCA, for conventional mean uptake (A), as well as four Haralick texture metrics (B-D). The correlation values were − 0.005, 0.33, − 0.36, − 0.31 and − 0.31. The more affected caudate side is shown, for PD subjects (n = 85).

Fig. 6 depicts correlations against the cognitive MoCA scale in PD subjects. Again, it was seen that conventional mean uptake did not show correlations (p-value = 0.96). At the same time, a distinct set of Haralick textural metrics, than those that were related to disease duration or motor scores, were seen to show significant correlations with the cognitive scale. Specifically, correlation, variance and cluster tendency depicted p-values of 0.0021, < 0.001, and 0.0034 respectively, while entropy depicted p-value of 0.0053, all of which were statistically significant after FDR adjustments. This finding also implies that the texture metrics are not merely disease-sensitive, but may also have specificity towards tracking of particular neuropsychological function that is impaired and associated with striatal dopamine degeneration. We also note that the fact that lower MoCA scores indicate poorer performance is consistent with the observed correlations with increased heterogeneity in tracer uptake. All the various p-values are summarized in Table 1.

Next, to extract independent factors, we performed multivariate analysis (as described in Section 2.4), and the results are also summarized in Table 1. In multivariate analysis of correlation against UPDRS scores, the Haralick textural feature agreement was retained as statistically significant (p-value < 0.001). In analysis of disease duration, depending on analysis of DD-diag. vs. DD-sympt., the single feature retained was contrast (p-value < 0.001) vs. homogeneity (p-value = 0.019), respectively. In the case of MoCA, variance was retained (p-value < 0.001), while age (which was not significant in univariate analysis; p = 0.076) became significant (p-value = 0.012).

Overall, it is worth noting that in multivariate analysis, only one of the texture features retains significance (even in the case of MoCA, the other significant predictor is age, not another texture metric). This we believe is related to the correlated nature of subsets of Haralick texture measures: similarly, in some oncology literature, there appears convergence towards the use of very few Haralick texture measures such as entropy and dissimilarity (e.g. (Hatt et al., 2015)). Our exploratory study here, first of its kind, also suggests that use of one or two Haralick texture measures can be sufficient, homogeneity or agreement, when correlating against UPDRS or disease duration (both DD-diag. And DD-sympt.), and variance when correlating against MoCA, in addition to age.

## 4 Discussion

The proposed framework can have important implications. Potentially enhanced sensitivity to track subtle neurochemical changes can provide novel insights into the relationship between dopaminergic alterations and PD clinical manifestations, while extending the clinical usefulness of this imaging technique. Furthermore, since there is strong evidence that DAT binding is reduced in the prodromal stage of PD (Nandhagopal et al., 2008, Sossi et al., 2010), these techniques can be applied to images from subjects at increased risk of PD (e.g. mutation carriers or subjects with rapid-eye-movement sleep behavior disorder) in an attempt to discern dopaminergic patterns that might be involved in pathogenesis and to assess the impact of novel disease modifying therapies. Importantly, the correlations between clinical, motor and neuropsychological measures are enhanced by applying the proposed methodology.

An exponential decline of tracer uptake in PD has been reported (Nandhagopal et al., 2009, Nandhagopal et al., 2011) for radiotracers of presynaptic dopaminergic integrity, including methylphenidate (MP), a PET marker for the membrane dopamine transporter (DAT) as also targeted in DAT SPECT. However, this was for an extensive time span from healthy state to PD. At the same time, to track state of disease within PD, a narrower, linear range is likely sufficient. In fact, our application of logarithmic operation to tracer uptake, or use of alternative correlations (e.g. Spearman) did not provide enhanced correlation with motor and cognitive symptoms.

To enhance conventional analysis, we also explored application of the concept of laterality (Salimpour and Shadmehr, 2014), utilized in the present work to assess asymmetry in tracer uptake. We quantified laterality via the definition | R-L |/((R + L)/2), wherein mean uptake information from the right (R) and left (L) sides of the structure of interest were utilized. However, this information, though useful for diagnostic purposes, did not enhance correlations with disease progression, relative to our proposed framework.

We found the caudate to provide significantly greater correlation of image-based texture metrics with clinical measures, compared to the putamen. Pathophysiologic studies of dopamine loss have clearly indicated rostrocaudal and dorsoventral gradients in the caudate and the putamen (see Fig. 1 in Kish et al., 1988). However, since dopamine loss is significantly greater in the putamen, which renders it suitable for classification/diagnosis, use of the caudate instead may be a valuable choice for enhanced tracking of disease, especially as combined with texture metrics that capture variations in the higher uptake caudate region. An analogous argument may be applied to prefer the use of the *less* affected putamen for tracking of disease, since following initial asymmetric loss of uptake in PD, it can provide a wider dynamic range (e.g. see Figs. 2–3 in (Nandhagopal et al., 2009)). We detected some improvements in performance when utilizing the less affected side of the putamen than the more affected side, though only for DD-sympt. and MoCA (not shown). However, these were significantly overshadowed by the strong findings in the caudate, wherein we found the more affected side to provide the greatest correlations with clinical measures, especially DD-diag. and MoCA.

The difficulties and uncertainties with PD diagnosis and disease metrics are well known and considerable. Early disease diagnosis remains a major challenge, since early symptoms may be subtle and nonspecific. The insidiousness of the onset is also responsible for why patients' ability to detect the first symptoms is greatly varied – affected by personality, level of education and professional background, the type of initial symptom (e.g. tremor versus bradykinesia), and likely a number of additional factors. The somewhat subjective nature of UPDRS evaluation makes this scale also prone to inter-rater variability. There have been multiple attempts to improve the reliability and accuracy of disease metrics and establishing early diagnosis, such as feature extraction algorithms using MRI data (Noh et al., 2015, Singh and Samavedham, 2015), population-based modeling using a combination of genetic and clinical data (Nalls et al., 2015) or combination of DAT SPECT and clinical data (Suwijn et al., 2015). Despite this, though we recognize uncertainties associated with onset (both time of diagnosis and time of first reported symptom) and disease metrics, the present framework with image-driven textural features had to rely on standard and validated data such as UPDRS and best available date of first symptom/diagnosis. In any case, it was observed for these metrics that significantly enhanced correlations were obtained with image-driven textural features in our proposed framework.

We are presently extending our investigation in a number of directions. Patterns of dopamine depletion in the basal ganglia are heterogeneous and more pronounced in the posterior putamen, which might be related to asymmetric impairment of dopaminergic neurons in the substantia nigra (Kish et al., 1988). For the caudate nucleus as well, the depletion of dopamine is heterogeneous, with more reduction in the most dorsal rostral region (Kish et al., 1988). PD also involves decreased connectivity from the more affected putamen to the cerebellum and contralateral putamen (Piggott et al., 1999), and the impaired striatum-cerebellar connection is likely a reflection of abnormal signals from the basal ganglia to influence cerebellar function (Bostan et al., 2010). We are investigating (Salimpour et al., 2015) application of a non-rigid normalization framework, wherein all structures of interest as imaged using DAT SPECT are registered to a common template via accompanying MRI data, enabling further investigation as to the extent by which different sub-regions *within* the putamen and caudate are correlated with and linked to different clinical symptoms.

This work included analysis of baseline scans in the PPMI dataset (first visit scans). Our ongoing efforts also include extension of this work to longitudinal scans. One issue to note is that the advanced metrics used in this work achieved significant correlations in the *more* difficult case of using cross-sectional data (since in longitudinal analysis, each subject is in a sense used to normalize itself and to compensate for inter-subject confounding factors). It is interesting to investigate to what extent intra-subject longitudinal analysis improves applicability of textural features to track progression of disease at an individual subject level.

Finally, we plan to extend our analysis of texture features (radiomics) to radiogenomics (also known as imaging genomics) (Hariri and Weinberger, 2003, Kerns et al., 2014, Medland et al., 2014), in which genetic information is additionally incorporated along with image-based textural features to find inter-relationships and to enable enhanced tracking of disease progression.

## 5 Conclusion

A number of Haralick textural features considered to characterize tracer uptake in DAT SPECT were found to depict significant correlations with clinical measures of UPDRS and disease duration. A different set of metrics was also seen to depict correlations with the cognitive MoCA scale. Overall, our results demonstrated ability to capture valuable information using advanced texture metrics, beyond conventional mean uptake analysis. Textural features as such may hold considerable potential as biomarkers of PD progression. Further longitudinal studies are needed to substantiate these findings.

## Conflicts of interest

None of the authors report any conflict of interest.

## Figures and Tables

**Table 1:** Univariate (U) and multivariate (M) analysis of correlation between textural features and clinical scores in the More affected caudate.

Parameters	p-values							
	UPDRS III		DD-diag.		DD-sympt.		MoCA	
	U	M	U	M	U	M	U	M
Conventional	0.94^a^		0.88^a^		0.34^a^		0.96^a^	
Entropy	0.0092		0.0027		–		0.0053	
Homogeneity	< 0.001		< 0.001		0.019^b^	0.019	–	
Agreement	< 0.001	< 0.001	0.0012		0.029^b^		–	
Dissimilarity	0.0022		< 0.001		0.021^b^		–	
Contrast	0.0085		< 0.001	< 0.001	0.040^b^		–	
Energy	0.031^b^		0.037^b^		–		0.041^b^	
MaxProbability	0.029^b^		–		–		–	
SumMean	–		–		–		0.029^b^	
Correlation	–		–		–		0.0021	
Variance	–		–		–		< 0.001	< 0.001
Cluster Tend.	–		–		–		0.0034	
Age	–		–		–		–	0.012

Notes:

(1) Two other Haralick features (cluster shade, inverse variance) were also included in the analyses, but had insignificant contributions, and thus are not shown in this table.

(2) We report p-values in the table, while those in univariate analysis that become non-significant after correction for multiple testing (FDR) are indicated using b (see below).

a p-Values for conventional normalized mean uptake were insignificant.

b p-Value < 0.05 but not significant after correction for multiple testing (p-values > 0.05 are not shown except for conventional analysis).
